# The European Union Emissions Trading System might yield large co-benefits from pollution reduction

**DOI:** 10.1073/pnas.2319908121

**Published:** 2024-07-01

**Authors:** Piero Basaglia, Jonas Grunau, Moritz A. Drupp

**Affiliations:** ^a^Department of Economics, University of Hamburg, 20146 Hamburg, Germany; ^b^Center for Earth System Research and Sustainability, 20146 Hamburg, Germany; ^c^Bordeaux School of Economics, University of Bordeaux, 33600 Pessac, France; ^d^Max Planck Institute for Meteorology, 20146 Hamburg, Germany; ^e^Hamburg Center for Health Economics, University of Hamburg, 20146 Hamburg, Germany; ^f^CESifo, 81679 Munich, Germany; ^g^Department of Economics, University of Gothenburg, 405 30 Göteborg, Sweden

**Keywords:** carbon markets, air pollution, EU ETS, policy evaluation, public health

## Abstract

Mitigating greenhouse gas emissions and reducing air pollution represent two pressing and interwoven environmental challenges. While international carbon markets, such as the European Union emissions trading system (EU ETS), have demonstrated their effectiveness in curbing carbon emissions (CO_2_), their indirect impact on hazardous co-pollutants remains understudied. This study investigates how key toxic air pollutants—sulfur dioxide (SO_2_), fine particulate matter (PM_2.5_), and nitrogen oxides (NO_x_)—evolved after the introduction of the EU ETS with a comparative analysis of regulated and unregulated sectors. Leveraging the generalized synthetic control method, we offer an ex post analysis of how the EU ETS and concurrent emission standards may have jointly generated sizable pollution reductions in regulated sectors between 2005 and 2021. We provide an aggregate assessment that these pollution reductions could translate into large health co-benefits, potentially in the hundreds of billions of Euros, even when bounding the effect of emission standards. These order-of-magnitude estimates underscore key implications for policy appraisal and motivate further microlevel research around the health co-benefits of carbon abatement.

The European Union emissions trading system (EU ETS) has been the cornerstone of European climate policy for almost two decades and represents the world’s largest supranational market-based climate policy instrument. It aims at curbing greenhouse gas emissions, primarily CO_2_, by setting an upper limit on carbon emissions in regulated sectors that declines over time. While the EU ETS does not directly regulate the emission of air pollutants, such as sulfur dioxide (SO_2_), fine particulate matter (PM_2.5_), and nitrogen oxides (NO_x_), these primarily result from fuel combustion (1) and thus as co-pollutants of CO_2_. As the EU ETS has proven effective in reducing CO_2_ emissions across regulated sectors (2), it is reasonable to expect that its impact likely extends to air pollutants. While simulation models predict that decreased reliance on fossil fuels could yield sizable health benefits (3), it remains unclear to what extent such benefits actually materialize. Yet, documenting the effects of real-world policy mixes on curbing harmful air pollution with ex post observational studies is key for informing policy.

This paper offers insights from quasi-experimental methods on the complementary impact of the EU ETS, along with concurrent emission standards for combustion, to reduce the emission of hazardous air pollutants—the prime environmental cause of global morbidity and mortality (1). We build on ref. 2 in using the generalized synthetic control method (GSCM) (4) to generate counterfactual pathways not for CO_2_, as ref. 2, but for SO_2_, PM_2.5_ and NO_x_ emissions with a weighted combination of unregulated sectors. This approximates counterfactual scenarios without the EU ETS, wherein concurrent policies (e.g., emission standards) would have maintained the same effect on emissions as before 2005. While concurrent regulations are a key obstacle for isolating individual policy effects, which the GSCM using aggregate-level data cannot fully disentangle, we provide an empirical assessment that pollution reductions likely due to the EU ETS might translate into large health co-benefits, even when bounding the effects of standards for large combustion plants (LCPs).

## Results

**Fig. 1.**
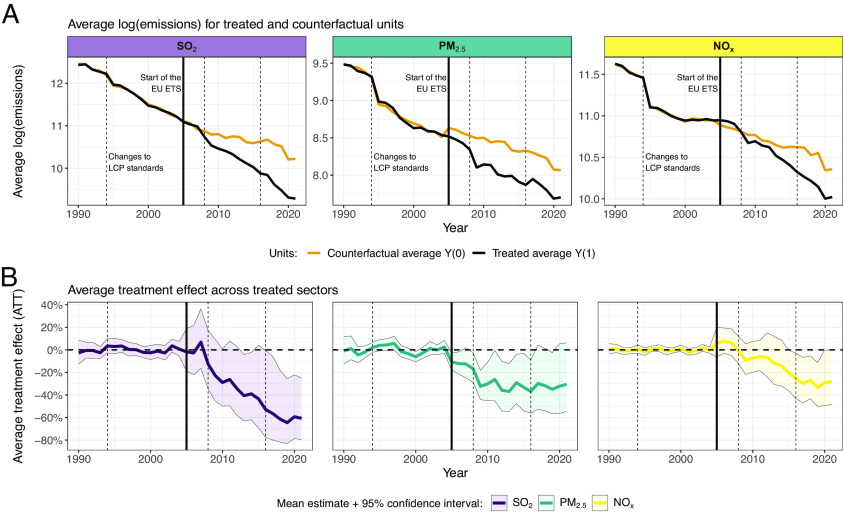
Differences in the evolution of air pollutant emissions by treatment status, capturing the joint effect of the EU ETS and concurrent policies, such as standards for LCPs. *Top* panels (*A*) display emissions paths for observed (black lines) and counterfactual (orange lines) emissions of key air pollutants—SO_2_, PM_2.5_, and NO_x_—aggregated by treatment status and averaged across countries. The vertical black line in the year 2005 reflects the start of the first trading period of the EU ETS. The vertical dashed lines indicate changes in EU directives on LCP standards, see *SI Appendix*, *Concurrent Policies* for details. The *Bottom* panels (*B*) show the estimated ATT of EU ETS regulated sectors due to the EU ETS and concurrent policy changes in percentage terms (solid lines) and 95% CI (shaded area) based on 1,000 bootstrapping iterations following ref. 4.

Fig. 1 shows pollution reductions since the start of the EU ETS in our main GSCM specification based on ref. 2. The *Top* panels (*A*) plot the path of SO_2_, PM_2.5_, and NO_x_ emissions in ETS-regulated sectors (black lines) and in synthetic counterfactuals (orange lines) from 1990 to 2021. The overlap between the black and orange lines before 2005 captures the pretreatment fit achieved by the GSCM. The same visual comparison post-2005 indicates pollution differences by treatment status, capturing the joint effect of the EU ETS and changes in concurrent policies. The *Bottom* panels of Fig. 1*B* plot the corresponding estimated average treatment effects on the treated (ATT) as solid lines. The shaded areas represent bootstrapped 95% CI, using 1,000 iterations (4).

**Fig. 2.**
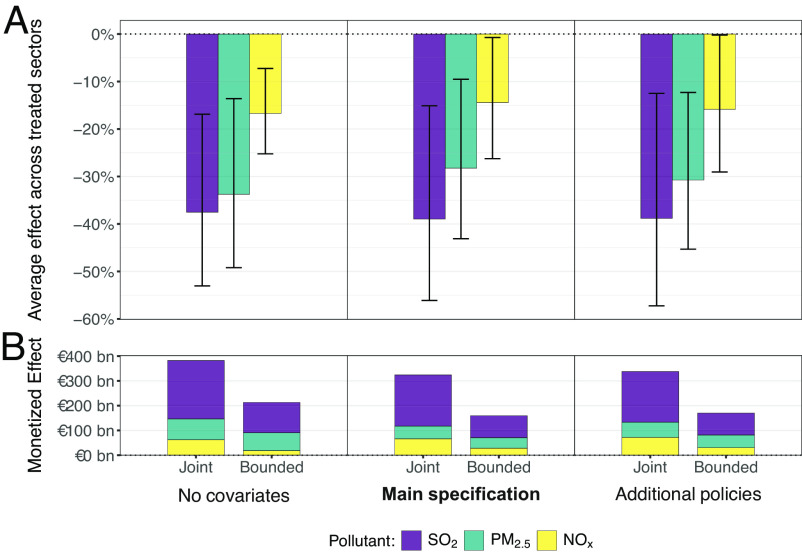
Estimated mean pollution reductions with 95% CI (*A*) and monetized aggregate health co-benefits (*B*) associated with treatment status, capturing the joint effect of the EU ETS and LCP emission standards (i.e., *Joint*). Bars denoted as *Bounded* in Panel (*B*) subtract the estimated benefits from emission reductions of LCPs jointly regulated by the ETS and standards to bound the direct impact of the latter on aggregate benefits (see *SI Appendix* for details). Health benefits are computed using pollutant-specific EU-wide cost estimates provided by the German Environment Agency, adjusted for inflation (*SI Appendix*). Model specifications: In contrast to *No covariates*, *Main Specification* includes log(GDP) and squared log(GDP) as in ref. 2; *Additional policies* also includes a binary carbon pricing indicator and log(Renewable electricity production), see also ref. 2. The *SI Appendix* offers estimations until 2016 for direct comparability with ref. 2.

Fig. 2 shows average annual reductions of air pollutants with 95% CI for the joint effect of the EU ETS and tightened concurrent policies in Panel (*A*) and gauges associated health benefits from the start of the EU ETS in Panel (*B*) by applying aggregate EU-wide cost estimates for SO_2_, PM_2.5_, and NO_x_ emissions from the German Environment Agency (*SI Appendix*). For the main specification following ref. 2, pollution changes amount to around −39% [−56%, −15%] for SO_2_, −28% [−43%, −10%] for PM_2.5_, and −14% [−26%, −1%] for NO_x_, averaged across units and periods. Plausible mechanisms for higher co-pollution decreases relative to CO_2_ may include fuel switching (5). Our estimates imply that the EU ETS and standards have jointly decreased 15.2 million tons of SO_2_, 0.9 million tons of PM_2.5_, and 4.8 million tons of NO_x_, which is equivalent to 18.3%, 3.3%, and 2.6% of observed economy-wide emissions from 2005 and 2021. Panel (*B*) also offers back-of-the-envelope calculations to bound the direct impacts of concurrent LCP standards by subtracting estimated emission reductions of LCPs jointly regulated by the EU ETS and standards (see *Joint*). While future microlevel analyses may more precisely disentangle overlapping policies, our *Bounded* aggregate assessment still suggests that the EU ETS might yield health co-benefits in the hundreds of billions of Euros.

We complement our main specification drawn from ref. 2 with alternatives that i) omit control covariates and ii) include additional covariates to address potential effects of concurrent policies on emissions (cf., Fig. 2). We provide further corroborating analyses in *SI Appendix* that i) include other covariates to explicitly control for residual key confounders, such as fuel prices, ii) report sensitivity analyses such as leave-one-out tests and in-time placebos, iii) consider different time frames for the posttreatment period, iv) use alternative emissions data, v) employ a complementary matrix-completion algorithm to yield counterfactuals, and vi) leverage a synthetic difference-in-difference estimator.

## Discussion

Our analysis suggests the world’s largest supranational carbon market likely leads to large reductions in air pollution and health damages, even when bounding the potential effects of EU emission standards. This has several implications.

First, a sole emphasis on CO_2_ reductions, as common in the literature evaluating carbon pricing schemes (e.g., refs. 2, 6, and 7), might severely underestimate the potential of climate policies to yield social benefits. A comprehensive policy evaluation should thus consider benefits beyond carbon abatement, such as reduced health damages through enhanced air quality (8).

Second, accounting for and communicating these health co-benefits, which deliver more immediate benefits to those affected by higher carbon prices, may additionally play a pivotal role in garnering support for climate policies (9).

Finally, our finding is relevant for the evaluation of distributional effects. While carbon pricing often imposes disproportionate direct consumer costs on lower-income households (10), disadvantaged communities also often bear a higher burden of air pollutant exposure, making ETS-driven air quality improvements a potential avenue for mitigating environmental inequities ( 11). The overall distributional burden on poorer households might thus be less pronounced.

While we provide a ballpark estimate that the EU ETS and concurrent emission standards may yield large health co-benefits, our analysis rests on aggregate pollution reductions and assesses co-benefits using official governmental guidance on EU-wide average cost estimates. This entails the assumption that pollutants cause the same monetary damage regardless of where in Europe they are emitted. Future research should investigate effects using microlevel data and determine how pollution reductions are distributed across heterogeneous firms, sectors, and locations. Such disaggregated analyses (e.g., employing chemical transport models with plant-level data) promise to further disentangle effects of sector-specific overlapping policies and to allow considering nonlinearities in how pollution reductions translate into health co-benefits. This will also require more fine-scale pollution cost estimates. Together, future research promises to generate more refined estimates of the co-benefits of the EU ETS and other policies to inform debates on policy design.

## Materials and Methods

### Data.

We use official national inventories of sectoral-level emissions of air pollutants from the European monitoring and evaluation programme (EMEP), available as an annual panel dataset covering EU countries from 1990 to 2021. We follow ref. 2 and focus on EU-25 countries. Cross-country panel data on gross domestic product (GDP) and other carbon pricing initiatives are extracted from the World Bank, renewable electricity production (in GWh) from Eurostat, and emissions of LCPs subject to standards from the European Pollutant Release and Transfer Register (E-PRTR).

### GSCM.

We follow ref. 2 in leveraging the GSCM (4) as an empirical framework to assess the effects of the EU ETS, in our case on air pollutants. The aim is to produce order-of-magnitude aggregate estimates. The principle of the synthetic control method is that each observation in the control group receives a weight based on its ability to align the (weighted) control group with the treatment group. The GSCM further incorporates an interactive fixed effects (IFE) model, controlling for unobserved time-varying confounding factors, and accommodates multiple treated units. For our GSCM analysis, we assume that each outcome (y), i.e., emissions of SO_2_, PM_2.5_, or NO_x_, can be explained by a corresponding factor model of the form (4):

[1] $$\begin{array}{c} log\left(y_{\mathrm{it}}\right)=\tau_{\mathrm{it}}ETS_{\mathrm{it}}+X_{\mathrm{it}}^{\prime }\beta +\lambda_{i}^{\prime }F_{t}+\epsilon_{\mathrm{it}}, \end{array}$$

where t={1990,…,2021} corresponds to the time period and i represents units, where each country contributes one regulated and one unregulated unit, aggregated from the respective ETS or non-ETS sectors. The sets T and C contain ETS and unregulated units, respectively, while $N_{\mathrm{tr}}$ and $N_{\mathrm{co}}$ denote the number of units within each set. $ETS_{\mathrm{it}}$ refers to the binary treatment indicator and equals 1 for treated units in the posttreatment period (i.e., i∈T and $t\geq t^{\mathrm{ETS}}$; $t^{\mathrm{ETS}}=2005$). The main parameter of interest is $\tau_{\mathrm{it}}$, the heterogeneous treatment effect at time t. $X_{\mathrm{it}}$ and β are vectors of observed covariates and their parameters, respectively. $F_{t}$ and $\lambda_{i}$ correspond to vectors of unobserved time-varying latent factors and their unit-specific factor loadings. Finally, $\epsilon_{\mathrm{it}}$ refers to idiosyncratic shocks. We estimate ATTs in periods $t\geq t^{\mathrm{ETS}}$:

[2] $$\begin{array}{c} ATT_{t,t\geq t^{\mathrm{ETS}}}=\frac{1}{N_{\mathrm{tr}}}\sum_{i\in T}\tau_{\mathrm{it}}\;, \end{array}$$

$\tau_{\mathrm{it}}$ is equal to $Y_{\mathrm{it}}\left(1\right)-Y_{\mathrm{it}}\left(0\right)$, i.e., the difference between the observed outcome of unit i at time t and its counterfactual outcome. As $Y_{\mathrm{it}}\left(0\right)$ is by definition unobserved for treated units, we employ the three-step GSCM estimation (2, 4) to obtain the counterfactual:

[3] $$\begin{array}{cc} Y_{it}=X_{it}^{\prime }\beta +\lambda_{i}^{\prime }F_{t}+\epsilon_{it}, & i\in C, \end{array}$$

[4] $$\begin{array}{cc} Y_{it}=X_{it}^{\prime }\hat{\beta }+\lambda_{i}^{\prime }{\hat{F}}_{t}+\eta_{it}, & i\in T, \end{array}\;t<t^{ETS},$$

[5] $$\begin{array}{cc} {\hat{Y}}_{it}(0)=X_{it}^{\prime }\hat{\beta }+\hat{\lambda_{i}^{\prime }}{\hat{F}}_{t}, & i\in T, \end{array}\;t\geq t^{ETS}.$$

First, leveraging data on control units, Eq. **3** is estimated to retrieve the coefficient on the observed covariates, $\hat{\beta }$, and time-varying factors, ${\hat{F}}_{t}$. Second, the GSCM algorithms select factor loadings ${\hat{\lambda }}_{i}$ for treated units by minimizing the mean squared prediction error (MSPE) in pretreatment years (Eq. **4**). Third, it imputes the predicted counterfactual outcome ${\hat{Y}}_{\mathrm{it}}\left(0\right)$ utilizing $\hat{\beta }$, ${\hat{F}}_{t}$, and ${\hat{\lambda }}_{i}$ (Eq. **5**). The GSCM selects the number of factors, $F_{t}$, and their loadings, $\lambda_{i}$, based on a data-driven cross-validation algorithm, which limits arbitrariness and reduces risks of overfitting. Our main specification (c.f., ref. 2) includes log(GDP) and log(GDP)^2^ in the IFE model. With predicted counterfactual outcomes for treated units, we can estimate the ATT (cf., Eq. **2**):

[6] $$\begin{array}{c} {\hat{\mathrm{ATT}}}_{t,t\geq t^{\mathrm{ETS}}}=\frac{1}{N_{\mathrm{tr}}}\sum_{i\in T}\left[Y_{\mathrm{it}}\left(1\right)-{\hat{Y}}_{\mathrm{it}}\left(0\right)\right]. \end{array}$$

To construct confidence intervals, we apply the parametric bootstrapping scheme from ref. 4 based on 1,000 replications.

Prior reviews do not suggest a loss of competitiveness in ETS sectors and thus treatment spillovers through leakage effects (12). To bound the effects of standards, we gauge emission reductions ascribable to targeted LCPs after 2008 (cf., Fig. 1) combining data on their emissions ($E_{t}$) from the E-PRTR with our ATTs as follows: $Reductions_{t}=\frac{E_{t}}{1-ATT_{t}}-E_{t}$. A discussion of identification caveats, such as residual confounders and spillovers via electricity prices, and alternative empirical strategies, can be found in *SI Appendix*.

## Supplementary Material

Appendix 01 (PDF)

## Data Availability

Data and code have been published in Ref. 13.
