# Supplementary material for: The European Union Emissions Trading System might yield large co-benefits from pollution reduction
Source: Proc Natl Acad Sci U S A. 2024 Jul 1;121(28):e2319908121. doi: 10.1073/pnas.2319908121 (PMC11252810; doi:10.1073/pnas.2319908121)
Supplement: Supplementary file 1 — Appendix 01 (PDF) [file pnas.2319908121.sapp.pdf]

Supporting Information for

The European Union Emissions Trading System might yield large co-benefits from pollution reduction

Piero Basaglia, Jonas Grunau, and Moritz A. Drupp

Corresponding Authors:
Piero Basaglia, Jonas Grunau, and Moritz A. Drupp

This PDF file includes:

Supporting text
SI References

Supporting Information Text

Extended methods

Materials. In our main analyses of how the EU ETS and concurrently tightened emission standards have jointly affected air pollution and health damages, we draw on official national inventories of sectoral-level emissions of air pollutants from the European Monitoring and Evaluation Programme (EMEP). The inter-jurisdictional program began following the Convention on Long-range Transboundary Air Pollution (CLRTAP) in 1979, which was crafted to foster international collaboration in addressing transboundary air pollution issues. EMEP involves collaboration with a wide network of scientists and national experts who contribute to the systematic collection, analysis, and reporting of emission data (1).

Emission-related information in industrial sectors is predominantly sourced from facility-level reporting (1), such as from the European Pollutant Release and Transfer Register (E-PRTR). Relying on facility-level data provides the advantage that it accounts for the adoption of emission abatement technologies in different facilities over time through plant retrofitting (cf., 2). When facility-level information is not readily available, national inventories compute sector-specific emission levels using country-specific emission factors combined with activity data derived from official statistical sources (energy and production statistics, traffic volumes, population sizes, etc.) or by employing models, such as for road transport emissions (see Ref. 3).

Data is available in the form of a panel dataset covering each European jurisdiction on an annual basis, starting from the year 1990. National inventories thus begin prior to the implementation of the EU ETS in 2005, which allows us to construct a synthetic counterfactual calibrated on a sufficiently long panel of pre-treatment periods ($T_0 = 15$) in line with recommendations ($T_0 > 10$) from Ref. (4). Furthermore, our dataset extends until 2021, covering four distinct trading phases (see European Commission for details) of the EU ETS over 17 years. During this time frame, the price of emissions permits surged substantially, ranging from below 5 Euros, such as in January 2013, to exceeding 80 Euros per tonne of carbon dioxide equivalent (tCO_2e) by the end of 2021. This extended temporal breadth allows us to explore the gradual unfolding of the dynamic impacts of an ETS over a relatively long time horizon and with substantial variation in carbon prices, setting our analysis apart from existing studies focused on co-pollution that often concentrate on pilot phases of other national emissions trading systems (e.g., 5–7) or do not have comparably extended pre- and post-intervention time frames (e.g., 8).

Sectoral definitions are consistent with the nomenclature from the 2006 Intergovernmental Panel on Climate Change (IPCC) Guidelines for National Greenhouse Gas Inventories and its 2019 Refinement (available here) developed for reporting air emissions under the United Nations Framework Convention on Climate Change. Following Ref. (9) (see also here), we refer to the following sectoral definitions to identify ETS sectors: energy (1.A.1), metals (1.A.2.a; 1.A.2.b; 2.C), minerals (1.A.2.f; 2.A), paper (1.A.2.d), and chemicals (1.A.2.c; 2.B). All remaining sectors are considered non-ETS sectors and are used in the synthetic counterfactuals (see below). As domestic aviation (1.A.3.a) has been regulated by the EU ETS since 2012 (see Ref. 10), we present the results of a robustness check where aviation is excluded from the sample as opposed to being part of the control units in our GitHub repository, which yields qualitatively similar results (available here).

For our estimations, we aggregate emissions from EU ETS-regulated and unregulated sectors, respectively, within each European country. This level of aggregation reflects the unit of analysis in our empirical models. Following Ref. (9), our main analyses therefore focus on EU-25 countries and our sample consists of 25 treated and control units, respectively ($N_{tr} = N_{co} = 25$). As data on the United Kingdom is not available in the 2023 EMEP release (available here), we obtain data on the UK from the 2021 EMEP release (available here), which only runs until 2019. Cross-country panel data on gross domestic product (GDP) and population are extracted from the DataBank, maintained by the World Bank. To trace the introduction of carbon pricing schemes across jurisdictions in the sample over time, we refer to the Carbon Pricing Dashboard (available here), an initiative of the World Bank that builds on the data and analyses of the annual *State and Trends of Carbon Pricing report series* (for further details see here). Annual renewable electricity production (measured in GWh) by jurisdiction is obtained from Eurostat (available here). Data on coal and lignite-fired combustion plants in Europe come from the Global Energy Monitor (available here). Data on the market price of natural gas, oil, and coal in global commodity markets is obtained from the International Monetary Fund (available here), while data to construct country-level oil, coal, and gas shares of total energy supply comes from Eurostat. All the R codes to assemble the data have been deposited in an online GitHub repository.

The Generalized Synthetic Control Method (GSCM). We employ a quasi-experimental research design to provide order-of-magnitude estimates of the potential aggregate impacts of the EU ETS (11), including impacts of concurrently tightened EU-wide emission standards, complementing the analysis by Ref. (9), which focused on carbon emissions. Quasi-experimental approaches can help researchers to estimate the effects of an intervention (i.e., the EU ETS) when a randomized controlled experiment with a counterfactual group is practically unfeasible or ethically impossible (12); oftentimes, however, identification of the effects of singular policies is limited by treatment spillovers or overlapping, concurrent policies. Recent advancements in the quasi-experimental literature have opened new avenues for shedding light on the potential efficacy of single policy instruments, or policy packages, in achieving their intended objectives as well as unintended effects (11). Among these, synthetic control methods (SCMs) are seen as *"[...] arguably the most important innovation in the policy evaluation literature in the last 15 years"* (13).

The core principle underpinning the SCM revolves around weighting control units to resemble a treated unit before the intervention occurs. Each observation in the control group receives a weight based on its ability to align the (weighted) control group with the treatment group in the pre-treatment period (for a recent review paper on the use of standard SCMs, see Ref. 14). Achieving a strong pre-treatment match with the SCM facilitates the assumption of baseline quasi-randomization

after re-weighting, which is key to identifying the effects of treatment in settings outside the realm of conventional experimental conditions (15). The weighted combination of untreated units then serves as an arguably plausible counterfactual following the intervention, allowing for a comparative analysis of outcome developments in treated and control units and the retrieval of average treatment effects on the treated (ATT). The underlying identifying assumption is that outcomes in synthetic control and treatment units would have trended similarly in the absence of treatment, aligning with the pre-intervention trajectories. This also entails the assumption that any effects of overlapping policies remain the same before and after the treatment the analysis seeks to examine. In the context of our analysis, we cannot fully disentangle the effects of emission standards given our method and aggregate-level data (see section on *Concurrent policies* below), which is why we report our main results as capturing the joint effect of the EU ETS and concurrently tightened overlapping policies, first and foremost emission standards.

Our main empirical investigation leverages a GSCM (4, 16) based on a linear interactive fixed effects (IFE) model (17). We selected the GSCM over the standard SCM (14) to closely complement the related work by Ref. (9) and as the GSCM exhibits desirable features along several dimensions. First, the GSCM explicitly allows absorbing level differences and unobserved time-varying shocks with interactive fixed effects (IFE). This is particularly important in our setting as one potential threat to the identification of the effects of the EU ETS is shocks specific to ETS sectors. These unobserved shocks, which cannot be properly accounted for by either unit or time fixed effects alone, are modeled through the more flexible IFE, which absorb trends at the aggregated sectoral level (i.e., ETS vs. non-ETS sectors) in each country. The inclusion of IFE has the further advantage of absorbing any level differences in emission levels driven by cross-country systematic differences in emission reporting practices for national inventories, which might otherwise introduce random noise in the estimations (cf., 9). IFE also allows us to control for emission variations influenced by common factors, such as fluctuations in global demand and variations in fuel prices in global commodity markets. Second, by including relevant control variables, our IFE model can additionally account for time-varying covariates and explicitly capture heterogeneous influences of other policies across countries, such as the effects of other carbon pricing schemes on European economies and their emissions (cf., 17). Third, the GSCM enhances the interpretability of SCM results by providing uncertainty estimates conditional on observed covariates, such as standard errors and confidence intervals, to conduct statistical inference. A further advantage of the GSCM estimator is its built-in cross-validation scheme, which automatically selects the model specification, limiting arbitrariness and researcher bias while reducing the risks of over-fitting. Specifically, the GSCM built-in algorithms select factor loadings $\hat{\boldsymbol{\lambda}}_i$ for the treated units by minimizing the mean squared prediction error (MSPE) in the pre-treatment years (cf., Eq. [4] in the Report). The MSPE is the squared deviation of the outcome predicted by the factor model using the estimated values obtained in Eq. [3] from the observed value Y_{it} of the treated units (see *Materials and Methods* in the Report), averaged across pre-treatment years. Finally, a key difference is that the GSCM employs dimension reduction prior to re-weighting which implies that, unlike with the standard SCM, weights do not bear a direct interpretation (see Refs. (4) and (15) for further details on the GSCM).

To closely follow Ref. (9) and complement their analysis on carbon abatement with pollution abatement, our *Main Specification* includes log(GDP) and log(GDP)^2 as main variables to the IFE model. We report parametric bootstrapped 95% confidence intervals (95-CI) based on 1,000 iterations (4). We opt for the parametric approach over the non-parametric procedure employed in Ref. (9), aligning with the original methodological guidance from Ref. (4, 18), given the limited number of treated units (i.e., $N_{tr} < 40$). Results are robust to the inclusion of the following controls: log(GDP per capita), log(Population), log(Renewable electricity production in GWh), a binary carbon price indicator, log(Retired coal-fired capacity), and market prices of coal, oil, and gas. The sections below describe key identification threats and alternative GSCM specifications employed to assess the robustness of our empirical findings. We further employ different emissions data and harness alternative research designs based on state-of-the-art methods for causal inference in quasi-experimental settings.

Treatment spillovers. The carbon price introduced by the EU ETS can affect firms in at least two ways (19). First, ETS firms must acquire and surrender ETS permits for each tonne of CO_2 emitted in the previous year (*direct* ETS costs). Second, both ETS and non-ETS firms may also pay for the carbon price reflected in higher electricity prices (*indirect* ETS costs) due to electricity producers passing through the carbon price on to consumer prices (e.g., Refs. 20–22). Notably, the latter are ultimately mediated by the existence of long-term negotiated lower electricity price rates (e.g., through collective contracts) for key industries within European national electricity markets, for instance in France, Germany, and Italy (23). In our empirical context, we cannot disentangle the effects of *direct* and *indirect* costs, so the reported treatment effects throughout the Report should be interpreted as the overarching impact of treatment status, encompassing both direct and indirect channels. Arguably, higher indirect carbon costs are expected to exert a more pronounced impact, and thus more concentrated, on sectors with high electricity intensity, including steel, chemicals, paper, and mineral production, all directly regulated by the EU ETS (23). Yet, to the extent by which these indirect costs are sizable among non-ETS activities, estimates might be biased as the GSCM would be comparing ETS emissions to a counterfactual based on partly (indirectly) regulated sectors.

To address competitiveness concerns, compensation schemes (24) or exemptions (25) are frequently implemented in the EU to shield firms from increased electricity costs due to climate policy. For instance, the EU ETS allows countries to compensate electro-intensive firms for the indirect carbon costs embodied in electricity prices based on their output levels (26). In the European context, complementary compensation schemes have been found to largely mitigate the extent to which increased indirect carbon costs affect electro-intensive production (e.g., 27)—which are plausibly the most exposed to treatment spillovers via electricity costs. Relatedly, Ref. (28) does not find any differential response to the EU ETS through market-wide price increases in electricity (or other carbon-intensive inputs) between ETS and non-ETS firms. More broadly, compensation linked to output volumes has been shown to limit losses in the product market in other policy contexts (29, 30), further alleviating concerns on potential competitiveness losses in our setting. Additionally, under output-based compensation, theory predicts

that producers will not pass on the full carbon cost, bounding the expected scope for further competitive distortions (31).

Treatment spillovers may occur with the relocation of economic activity from ETS sectors to untreated sectors (or *leakage* effects), due to the former becoming less competitive due to higher carbon costs. While based on previous findings in the literature, we do not expect sizable competitiveness losses in regulated sectors due to the EU ETS (Refs. 28, 32–37), we do not directly test for and thus cannot fully rule out market-level spillovers due to the EU ETS.

Concurrent policies. A key caveat concerning the identification of the effects of the EU ETS on the emission of air pollutants—i.e., of an interpretation of the results that the effect of treatment status shown in Figures 1 and 2 in the Report solely captures the effects of the EU ETS—is the influence of other concurrent pollution-reduction policies, in particular EU-wide emission standards. The first EU Directive, within our estimation sample, on the limitation of emissions of certain pollutants into the atmosphere from large combustion plants (LCPs) dates back to 1988 (88/609/EEC). Specifically, the directive outlined maximum thresholds for the yearly average emission intensity of SO_2, NO_x, and dust (PM) to all LCPs with a rated thermal input of 50 MW or more. This implies a sizable regulatory overlap with the EU ETS, which covers emissions from combustion installations with a rated thermal input surpassing 20 MW (i.e., nearly all LCPs). Emission standards were subsequently amended in 1994 (Directive 94/66/EC)—highlighted as the first instance of LCP regulation changes during our time frame depicted in Figure 1 in the Report—and then revised again in October 2001 (LCPD 2001/80/EC), becoming effective in 2008. Under the latter, LCPs licensed before 1 July 1987 (henceforth *old* plants) had the choice to either adhere to the new emission limits (henceforth *compliance*) or opt out, which would limit them to 20,000 operating hours between 2008 and 2015 and require a shutdown afterward. Member States could also choose to comply by adopting a national emission reduction plan to limit aggregate emissions to the same levels that would have been achieved by applying the new standards to existing plants.

Thereafter, the Industrial Emissions Directive (IED 2010/75/EC) superseded the regulatory framework established by previous emission performance standards, turning effective in 2016 and requiring compliance by 2021 (or shutdown by 2023 with capped hours for those who opted out). The IED introduced somewhat more lenient enforcement mechanisms by (i) granting national regulators the authority to override compliance when they assess that local conditions entail "*disproportionately higher costs compared to the environmental benefits*" (Art. 15, para. 4a) and (ii) providing additional derogations - i.e., *small isolated systems* (cf., Art. 34) and *district heating plants* (cf., Art. 35). The revised binding limits were overall less ambitious than their predecessors (e.g., SO_2 limits were not revised) and were projected to result in limited further improvements, if any, in pollution intensity by 2021, as highlighted in an interim report by the European Environment Agency (EEA) (38).

The decrease in emission levels within the ETS sectors, relative to the averages in non-ETS activities, as depicted in Figures 1 and 2 of the Report, could be partially attributed to a diminished generation capacity in high-polluting LCPs driven by increasingly stringent regulatory standards (e.g., plant closures). Crucially, the incorporation of IFE in our GSCM specifications captures any heterogeneous impacts of emission standards on air pollutants across units before 2005, including early anticipatory behavior to their 2008 revisions (i.e., retrofit investments translating into lower emission intensity). This has two key advantages for our empirical approach. First, this holds particular relevance because of the interaction between 2008 emission standards and the Integrated Pollution Prevention and Control (IPPC) Directive (96/61/EC). The latter mandated early compliance with best available techniques, in line with upcoming more stringent standards, for all new combustion plants starting from October 30, 1999. We would therefore reasonably expect early compliance by newly constructed plants, which would be accounted for by the IFE. Second, if pre-treatment trends in air pollutants arising from the gradual roll-out of early emission standards (largely) persist after the start of the EU ETS, the IFE would, at least in part, mitigate the confounding impacts of post-2005 standard revisions. Yet, potential systematic shifts in the underlying effect of post-2005 standards would still introduce residual confounding variation.

We rely on insights gleaned from existing evaluations to provide some perspective on the expected effects of post-2005 standards on air pollution. To our knowledge, no peer-reviewed study has yet documented clear effects of EU-wide industrial air pollution standards on emissions. A working paper has examined EU emission standards and their effects on pollution intensity (cf., Ref. 39). These preliminary findings suggest that older, more polluting plants that chose compliance significantly reduced emission intensities from 2008 vis-a-vis opted-out plants. However, the findings also reveal that the policy contributed to delayed shutdowns for plants that opted out, resulting in prolonged highly polluting operations and that around 60% of opt-out plants actually continued to operate after 2015. This aligns with descriptive evidence from Ref. (40), which indicates that larger plants running on coal and lignite were more likely to be opted out. Hence, based on the balance of the limited empirical insights, it remains challenging to draw clear predictions on the net impact of the LCP directives on aggregate emissions.

To shed light on the potential impact of changes in emission standards on ETS sectors within our setting we proceed as follows. First, we narrow our focus exclusively to the power sector (UN code 1.A.1.a), as it was the main sector targeted by LCP emission standards (38). Our sectoral analysis demonstrates discernible declines in emissions occurring before the new LCP standards became binding in 2008 (especially for $PM_{2.5}$), reinforcing the attribution of part of the observed effects on air pollutants to the EU ETS (plot available here). In line with the findings from Ref. (39), we do not expect these results to be driven by anticipation of standards compliance from old plants. This is primarily due to the high retrofitting expenses for older plants and the absence of an IPPC requirement for combustion units operating pre-1999. Second, we test whether our results are affected by accounting for the annual retired capacity of the old coal-fired combustion fleet as an additional control to our main GSCM specification in the Brief Report (available here). We yield similar results to our *Main specification*.

Third, we put our findings into perspective concerning the potential effects of the LCPD and other concurrent energy-transition policies. To this end, we subtract reported emissions from old coal and lignite-fired power plants that announced their retirement between 2010 and 2017 (time frame constrained by data availability) from the estimated absolute pollution

reductions in our *Main Specification* using data collected by Europe Beyond Coal (cf., Ref. 41 for a similar approach). Assuming that the EU ETS had no influence on coal and lignite-fired power plants' closure, a back-of-the-envelope calculation yields a remaining cumulative decrease of around 13 million tons of SO_2, 0.8 million tons of $PM_{2.5}$, and 2.9 million tons of NO_x (due to lack of data, we use dust emissions to proxy $PM_{2.5}$). This suggests that the associated health co-benefits of the EU ETS would still be on the order of around 260 billion Euros. Further subtracting emissions from old operating plants that did not announce retirement plans lowers this value to around 220 billion Euros.

As an alternative approach, we retrieve emissions from polluting *thermal power stations and other combustion plants* (E-PRTR activity code 1c) from the E-PRTR since 2008 (first year when the LCPD became effective) to 2021. Micro-level data (only available from 2007 onward) allows us to isolate combustion-related emissions from installations with a rated heat input of 50 MW and thus subject to the 2008 LCPD (EC Regulation 166/2006). We then consider a hypothetical scenario where pollution reductions across large combustion plants are solely attributed to binding emission standards (and not at all to the EU ETS). Using E-PRTR plant-level data, we take a simplified approach combining the estimated pollutant-specific $\widehat{ATT}_{t,t\geq t^{ETS}}$ with observed emission levels after 2008. Specifically, we assume that without policy intervention, aggregate combustion emissions of pollutant (p) for each year (t) would have been higher by a percentage matching the evolution of ATTs in the *Main Specification*. In doing so, we can then approximate policy-driven reductions as: $Reductions_{p,t} = \frac{Emissions_{p,t}}{1-ATT_{p,t}} - Emissions_{p,t}$, which we, for the sake of argument, assume to be fully attributed to emission standards. Finally, by subtracting the cumulative damages of the estimated reductions from our benefit assessment based on the *Main Specification*, we derive a lower-bound approximation of health co-benefits of the EU ETS of around 160 billion Euros (see here for the replication codes). Acknowledging the potential influence of ETS carbon costs on decisions to retire power plants and, more generally, their activities (41), and considering that this back-of-the-envelope simulation considered all emissions from *thermal power stations and other combustion plants* jointly subject to emission standards but also to the EU ETS, we note that this approach could underestimate the effects of the latter. Because of their overlapping nature, the interactions between the EU ETS and emission standards cannot be treated in full isolation from each other in our sectoral-level study design based on Ref. (9). For instance, emission standards may, in turn, induce costs associated with having to install scrubbers, making fuel switching and different investment paths more likely under the ETS. Despite that, these additional insights support the interpretation that EU ETS might have led to likely economically sizable health co-benefits.

Future causal inference studies using plant-level data may further refine our estimates. However, the use of micro-data to address this issue also faces several data challenges, primarily due to a discontinuity in facility-level reporting during a transition across EU-wide data registries (the former EPER to the E-PRTR). Firstly, this transition coincided with the introduction of the EU ETS and the roll-out of the new 2008 LCP Directive announced in 2001, resulting in a data gap between 2004 and 2007 (EC Regulation 166/2006). Secondly, EPER reports data for the years 2001 and 2004 thus providing information only for two pre-ETS years. Thirdly, the transition to the E-PRTR also aimed to shift from locally or nationally agreed methodologies to a more internationally harmonized approach, on an EU-wide scale (Art. 5, para. 4). This poses empirical challenges in untangling policy effects from discontinuities in data collection, reporting practices, and sampling, which can be overcome by the use of harmonized sectoral-level inventories, albeit with a trade-off between data granularity and reporting consistency. Finally, the concentration of a substantial number of LCPs in Germany (17% of the total) compounds the issue, as Germany (together with Sweden) does not officially disclose the individual compliance status of its LCPs (42), adding a layer of complexity for micro-level evaluations focused on the effects of EU standards. While acknowledging limitations in both sectoral and micro-level approaches, our results offer a first aggregate assessment in a quasi-experimental framework, documenting that the EU ETS may have led to sizable aggregate-level co-benefits. Yet, one key limitation of our approach is that it does not allow for the estimation of spatial heterogeneous treatment effects across treated firms. While beyond the scope of this Report, future research leveraging alternative policy shocks and study designs based on micro-level data should delve deeper into the interplay between multiple instruments and their effects on plants' emissions. Future research should also focus on indirect channels, which we do not consider here, acknowledging how the early roll-out of air pollution standards might have created the political conditions to enact more stringent climate policies (e.g., EU ETS).

Putting the magnitude of our estimates into perspective, descriptive trends from the data used in our main specification reveal that emissions in ETS regulated sectors had declined by approximately 85% (82%) for SO_2, 57% (52%) for $PM_{2.5}$, and 63% (57%) for NO_x in 2021 (2019) relative to 2004-levels (see here for the relevant code and further descriptive plots). To get a glimpse of how these observed emissions reductions compare with estimated reductions, attributed to the EU ETS and concurrent policies, the ATT_{2021} (ATT_{2019}) is 61% (65%) for SO_2, 31% (35%) for $PM_{2.5}$, and 28% (33%) for NO_x (see Eq. 2 in the *Brief Report* for the calculation of ATT_t). This simple comparison provides a general indication that what the GSCM captures as the ATT of treatment status, which includes the joint effect of the EU ETS and overlapping policies, contributed only partly, albeit substantially, to the overall emissions reductions between 2005 and 2021. This implies that macroeconomic drivers and technological progress represent a substantial share of the overall decrease, particularly for $PM_{2.5}$ and NO_x.

Finally, in a parallel manner, as the counterfactual is built upon non-ETS sector emissions, which still had some level of regulation under EU-wide emission standards on transport emissions (43) or unilateral fuel taxes (44, 45), and the EU's Effort Sharing Decision, the effect of the treatment status identified with the GSCM, which we attribute to the EU ETS and concurrent standards, might also underestimate the true effect size, as it does not compare emission reductions against a no-policy scenario, in line with Ref. (9). In sum, while our empirical framework, which closely mirrors the analysis of Ref. (9), can generate a first ball-park estimate of the potential magnitude to which the EU ETS might have generated co-benefits due to pollution reductions, our results do not constitute precise, causally identified estimates and should be refined in future work.

Alternative GSCM model specifications. We test a number of alternative GSCM specifications of our main analysis, capturing the joint effect of the EU ETS and tightened concurrent policies, particularly EU-wide emission standards for LCPs, where we (i) explicitly account for the residual impacts of additional potential confounders after absorbing IFE; (ii) perform leave-one-out tests for our main specification where we iteratively exclude individual countries from the analysis; (iii) conduct placebo exercises where we shift the treatment before the start of the EU ETS in 2005; (iv) consider alternative time frames for the post-treatment period, and (v) employ a SCM estimator using an alternative matrix-completion (MC) algorithm from Ref. (46).

We begin by estimating additional model specifications where we include log(GDP) and $\log(\text{GDP})^2$ alongside IFE (as in the *Main Specification* model; see Figure 2 in the Report) together with further control variables, in line with Ref. (9). These variables include GDP per capita, population, renewable electricity production, and a dummy indicator for other carbon pricing policies (cf., *Materials* for more details). The rationale for including more covariates is twofold. The first two variables allow us to more precisely model the influence of economic and demographic factors, respectively, on air pollutant emissions. In contrast, the latter two controls capture the potential effect of other carbon pricing initiatives and any policy interventions that fostered a faster uptake of renewable energy production, operating at both EU and country levels. We test the robustness of our results when separately adding each of the four variables to the model of our main specification that only includes log(GDP) and $\log(\text{GDP})^2$. These analyses corroborate the findings presented in the Report and are available here.

We then consider the impacts of fuel price changes in our estimations. Data from the IMF highlights that oil, coal, and gas prices varied substantially since the start of the EU ETS (see here). This likely affects firms' fuel input choices and ultimately emissions. This effect, however, should be captured by the IFE model (Eqs. 3-5 in the *Brief Report*), as the latter models common factors varying across time ($\boldsymbol{F}_t$ in Eq. 3), such as fuel price developments due to movements in global commodity markets. Moreover, the IFE model estimates how these common factors impact treated units differently (e.g., due to systematic differences in energy infrastructure) through heterogeneous factor loadings ($\boldsymbol{\lambda}_i$) that are interacted with the time effects in Eq. 4 in a data-driven selection process (see also the section on the GSCM in this *SI Appendix*). While we expect the IFE model to largely absorb the effects of fuel prices on emissions, we introduce a specification in which we explicitly include oil, coal, and gas prices as covariates for additional robustness. To model the country-specific exposure to fuel-specific price changes, we multiply individual fuel prices by a country-specific fuel use share, which we derive from each fuel's average contribution to the country's pre-treatment total energy supply (from 1990 to 2004). Fuel prices are normalized, with 1990 serving as the base year (i.e., indexed to 100). The results are very similar to our main specification (see here), which reflects the similarity between how we construct the fuel price variables and how the IFE model deals with them. Thus, while our GSCM approach can capture the aggregate effects of fuel price changes on emissions, future micro-level analyses leveraging plant-specific fuel prices are expected to provide more refined estimations. However, to our knowledge, obtaining such fine-grained data harmonized at an EU-wide level is currently not feasible.

Furthermore, we perform a set of standard robustness checks from the SCM literature (14). First, we begin by conducting leave-one-out tests for our main specification where we iteratively exclude individual countries from the analysis to rule out that our results are driven by single outlier jurisdictions, following Ref. (47). We present the outcome of these tests in the GitHub repository and show that our results are robust across the different combinations of countries included in the analysis. These tests address identification concerns arising from unilateral policies affecting ETS sectors that could accelerate air emissions reductions at the national level (e.g., Ref. 41). Second, we perform an in-time placebo test where the treatment period (t^{ETS} in the *Generalized Synthetic Control* section in the Report) is moved to 2002, i.e., three years before the actual introduction of the EU ETS in 2005. The presence of a statistically significant and persistent placebo effect prior to the beginning of the ETS would raise concerns about the reliability of the findings presented in Figure 1 in the Report. Our results show that the synthetic counterfactuals closely mirror the observed emission trends in regulated sectors following the placebo treatment and no significant deviation is observed before 2005, thus strengthening the attribution of the detected effects on emissions to the EU ETS (or its treatment status). Results of the in-time placebo are available here in the online repository. We also present how the results change when shifting the beginning of the post-treatment period to 2008 as in Ref. (9), which coincides with the second trading period of the EU ETS and the end of the pilot phase (available here). We yield comparable results for SO_2. For $PM_{2.5}$, the ATT is significantly smaller when moving the treatment period t^{ETS} to 2008, implying that a sizeable share of the emissions reductions may have happened early on during the first ETS phase. The reductions in NO_x, on the other hand, seem to have occurred later on, as the ATT estimate gets larger when $t^{ETS} = 2008$

Finally, we complement our baseline GSCM estimations by employing a matrix-completion (MC) algorithm (46) building on the machine learning literature (11) to generate alternative synthetic counterfactuals (see Ref. 48, 49 for similar approaches). MC operates by treating counterfactual, untreated observations within the treatment group as missing values in a matrix. These missing values are imputed through a regularized process that imposes penalties on matrix complexity. Specifically, following the notation in Ref (49), imputation is conducted by estimating a regularized representation (denoted as $\hat{L}$) of the original outcome matrix Y_{it}. This estimation aims to minimize the difference between observed and estimated outcomes. Unlike with the GSCM, synthetic counterfactuals are not derived by directly estimating factors and loadings. Instead, the MC algorithm solves the following minimization problem by introducing a penalty for matrix complexity using a regularization term, λ, determined through cross-validation (or *tuning parameter* in the machine learning literature) (15):

$$\hat{L} = \arg\min_{L} \left\{ \sum_{i,t \in \vartheta} \frac{(Y_{it} - L_{it})^2}{|\vartheta|} + \lambda ||\boldsymbol{L}||_* \right\} . \qquad [1]$$

The average treatment effect on the treated (ATT) is then estimated as the average difference between the observed (treated)

outcomes and the imputed (missing) untreated outcomes, $\hat{L}$. This approach preserves the flexibility to incorporate both time and unit fixed effects. Results based on MC, closely replicating our *Main Specification*, can be found here. We refer the reader to Ref. (46) and Ref. (13) for more background and methodological details on the MC algorithm and to Ref. (15) for a direct comparison of MC to other IFE estimators, including the GSCM. All the R codes used to conduct the aforementioned complementary analyses and the corresponding results have been deposited on GitHub.

Comparison to Bayer and Aklin (2020) and restriction to a pre-pandemic time frame. As we take the analysis in Ref. (9) as a comparative starting point for our main specification, we here compare their reported CO_2 reductions of 8.1% between 2005 and 2016 to our estimated reductions of 39% for SO_2, 28% for $PM_{2.5}$, and 14% for NO_x between 2005 and 2021. Before aligning the time frames of our studies, we note that reductions of CO_2 and co-pollutants are not necessarily expected to decrease in a ratio of 1:1 ex-ante. More specifically, while we do not investigate the mechanisms that drive our estimated emissions reductions, several studies indicate that short-term operational fuel switching—i.e., from coal to gas—could be one among many relevant mechanisms in the context of the EU ETS (e.g. Refs. 50–52). Replacing coal with gas generates substantially larger relative reductions in SO_2 and particulate matter than for CO_2 (53, 54). This potential mechanism could imply that estimates for SO_2 and $PM_{2.5}$ are expected to be larger than those for NO_x and CO_2.

To enhance the comparability of our analysis to the one in Ref. (9), we further set the end of our post-treatment period to 2016 as in Ref. (9) instead of 2021 (results available here). The estimates for the three air pollutants decrease relative to our main specification, yet the central estimates of reductions in SO_2 and $PM_{2.5}$ are still substantially larger than for CO_2.

Finally, to address concerns about the impacts of the COVID-19 pandemic in 2020 and 2021, we exclude these two years in an additional specification. The results (available here) demonstrate that the estimated reductions, albeit smaller than in the main specification, are in line with our main conclusion that the EU ETS, in conjunction with overlapping policies, contributed to substantial pollution reductions.

Alternative emissions data. To corroborate our findings, we examine the qualitative robustness of our estimations when utilizing alternative emissions data from Ref. (55), which are independently maintained by a non-governmental source. This mitigates concerns that effects may be partially driven by potential biases or underreporting of emissions influenced by political considerations (56). The data stems from the Emissions Database for Global Atmospheric Research (EDGAR), which is a global database of anthropogenic emissions of greenhouse gases and air pollutants, spanning—as of November 2023—from 1970 to 2018 (2022 for greenhouse gases) compiled by the EU Joint Research Centre (JRC). Emissions are computed relying on a consistent technology-based emission factor approach and harmonized sector definitions in line with IPCC guidelines.

One limitation that further motivates our use of EMEP data in our primary analyses is that EDGAR data offers lower sectoral granularity data, particularly for manufacturing sectors , where 1.A.2 is the most disaggregated sectoral level available. As explained in the *Materials* section of this document, the EU ETS indeed covers most sub-sectors of 1.A.2 (e.g., 1.A.2.a: iron and steel). However, following Ref. (9), 1.A.2.g is not considered to be regulated, yet it represents a large share of manufacturing emissions. It follows that considering the whole sector 1.A.2 as treated—or untreated—would introduce measurement errors in identifying treated and control sectors and ultimately bias our estimations.

To circumvent this data constraint, we use EMEP data on the sub-sectors of 1.A.2 to calculate the share of each sub-sector within 1.A.2 for each country-year combination. We then employ these shares to split the EDGAR data on 1.A.2 into the sub-sectors of 1.A.2. This allows us to enhance the precision of emissions identification within 1.A.2 sectors, leveraging information at more detailed sectoral codes to distinguish between ETS and non-ETS sectors. The R code that executes these steps is part of the GitHub repository (files '/src/R/functions.R', '/src/R/data_prep.R', '/src/R/gscm_set_up.R').

Re-estimating our *Main Specification* leveraging EDGAR data (up to 2018 only, which is the latest year currently available), we provide complementary evidence that corroborates our main GSCM findings. For SO_2, the estimates are very similar when using EDGAR data, while they decrease for $PM_{2.5}$ and increase for NO_x. More specifically, we estimate average annual changes which amount to around −28% for SO_2 (95-CI = [−44%, −8%], P = 0.009), −19% for $PM_{2.5}$ (95-CI = [−31%, −5%], P = 0.010), and −30% for NO_x (95-CI = [−37%, −21%], P = 0.000). The results are available here.

Alternative methods. We examine the robustness of our results by drawing on alternative, more recent methods for ex-post policy evaluations. To this end, we employ the synthetic difference-in-differences (SDID) methodology, which combines desirable features of SCMs and a two-way fixed effects (TWFE) difference-in-differences (DID) approach (57). Drawing on Ref. (57), this section describes how we employ the SDID estimation procedure in our empirical setting and contrast its relative strengths relative to a TWFE-DID approach in addressing potential bias in the identification of effects.

As an input requirement, the SDID estimator analysis requires a balanced panel of N units or groups, observed over T time periods. We consider a setup where a subset of N is regulated (i.e., subject to the EU ETS) as indicated by a binary treatment indicator, which we denote by ETS_{it}. The indicator ETS_{it} equals one if unit i at time t is regulated by the EU ETS and equals 0 otherwise. In this setting, the SDID estimation of the ATT (denoted by τ^{sdid}) can be written as follows (57):

$$\left(\hat{\tau}^{\text{sdid}}, \hat{\mu}, \hat{\alpha}, \hat{\beta}\right) = \arg\min_{\tau,\mu,\alpha,\beta} \left\{ \sum_{i=1}^{N} \sum_{t=1}^{T} \left(Y_{it} - \mu - \alpha_i - \beta_t - ETS_{it}\tau\right)^2 \cdot \hat{\omega}_i^{\text{sdid}} \cdot \hat{\lambda}_t^{\text{sdid}} \right\}, \qquad [2]$$

where the parameter $\hat{\tau}^{\text{sdid}}$ is the ATT estimated in a TWFE regression with optimally chosen unit-specific ($\hat{\omega}_i^{\text{sdid}}$) and time ($\hat{\lambda}_t^{\text{sdid}}$) weights. The underlying intuition behind the inclusion of $\hat{\omega}_i^{\text{sdid}}$ and $\hat{\lambda}_t^{\text{sdid}}$ is that *unit-specific* weights allow to yield

matching pre-intervention trends while *time-specific* weights are introduced in the regressions to reduce the influence of time periods that significantly differ from post-treatment periods and increase model precision. Crucially, matching pre-intervention trends are pivotal to assuming quasi-randomization (after re-weighting) in the identification of causal effects (see Ref. 57 (Section 2.1, Algorithm 1) for details on the estimation of weights). In essence, the SDID estimator can be seen as a DID analysis with weighted observations. This approach allows the SDID estimator to sidestep some of the typical issues of standard DID and SCM. These include the inability to estimate causal relationships when the parallel trends assumption is not satisfied for DID and the requirement in SCM for the treated unit to be located within a *convex hull* of control units.

The inclusion of unit fixed effects (α_i) implies that the SDID estimator, by choosing unit weights ($\hat{\omega}_i$), will match treated and control units based on pre-treatment trends, as the fixed effects (α_i) absorb any level differences (i.e., similar to the GSCM). The presence of time effects (β_t) allows for common temporal aggregate factors. In our setting, the latter refers to underlying trends or fluctuations in emissions that occur due to factors such as technological advancements, economic conditions, or other external drivers that change emissions levels over time independently of the EU ETS or standards.

In contrast, the corresponding standard TWFE-DID procedure essentially mirrors the SDID regression but assigns equal weights to all time periods and units, making diverging pre-treatment trends between treated and control units a potential threat to identifying causal effects. That is, TWFE-DID estimates will be biased when unobserved time-varying confounders exist, as we cannot plausibly assume that outcomes in control and treatment groups would have trended similarly in the absence of treatment (11). The TWFE-DID estimation of the ATT (denoted by τ^{did}) can be written as follows:

$$\left(\hat{\tau}^{\text{did}}, \hat{\mu}, \hat{\alpha}, \hat{\beta}\right) = \arg\min_{\tau,\mu,\alpha,\beta} \left\{ \sum_{i=1}^{N} \sum_{t=1}^{T} \left(Y_{it} - \mu - \alpha_i - \beta_t - ETS_{it}\tau\right)^2 \right\} . \qquad [3]$$

While variants of the latter approach (i.e., TWFE-DID in Eq. [3]) have been employed by recent studies that have investigated the pilot Chinese ETS (e.g., 6, 58, and 59), we depart from these studies by employing several more flexible quasi-experimental methods (GSCM, MC algorithm, SDID). The methods we draw on formally account for and are robust to diverging pre-treatment trends between treated and control units by introducing unit-specific weights, thus reducing bias in the estimations. The SDID further improves model precision by disregarding pre-treatment periods that exhibit substantial differences from the post-treatment periods (i.e., with time-specific weights), making the SDID estimator doubly robust to both diverging pre-trends and model misspecification (15, 57). Finally, in contrast to other approaches to address divergent pre-trends in a TWFE-DID estimation, such as one-to-one matching with propensity scores (see Refs. 5 and 7 for examples), our methods avoid discarding non-matching observations. Instead, our estimation strategies assign them lower unit-specific weights, allowing us to retain a larger estimation sample and hence greater statistical power for inference. To further condition our estimations from Eq. [2] on time-varying covariates, we apply the SDID algorithm to the residuals computed as:

$$Y_{it}^{\text{res}} = Y_{it} - X_{it}\hat{\beta} \, , \qquad [4]$$

where $\hat{\beta}$ is calculating by regressing Y_{it} on X_{it}. We can then conduct inference by constructing confidence intervals for the treatment effect leveraging the bootstrap procedure described in Ref. (57, Algorithm 2). Our confidence intervals are estimated based on 800 replications. Finally, to account for dynamic variations in the relationship between covariates and the outcomes among treated and control units, we additionally apply the optimization procedure described in Ref. (60) and (61).

We show how our estimations change when employing both the preferred SDID (Eq. [2]) and TWFE-DID (Eq. [3]) estimators to fit our *Main Specification* (i.e., including log(GDP) and log(GDP)^2 as control variables). Both estimation approaches point to sizable reductions in co-pollutants. However, our SDID estimates consistently yield more conservative results relative to TWFE-DID, indicating the potential for bias in observational studies that do not account for diverging pre-treatment trends. The SDID estimator yields average annual changes of around −19% for SO_2 (95-CI = [−36%, 3%], P = 0.091), −28% for $PM_{2.5}$ (95-CI = [−43%, −9%], P = 0.003), and −12% for NO_x (95-CI = [−22%, 1%], P = 0.064). The TWFE-DID estimator, in contrast, yields average annual changes which amount to around −29% for SO_2 (95-CI = [−47%, −5%], P = 0.020), −39% for $PM_{2.5}$ (95-CI = [−54%, −19%], P = 0.001), and −24% for NO_x (95-CI = [−36%, −9%], P = 0.003).

Crucially, one difference between our SDID and GSCM results lies in the requirement for a balanced sample to employ the SDID estimator. This implies that, in our SDID estimations, we need to drop countries for which we do not have balanced observations throughout the entire estimation period (i.e., 1990 - 2021). These include most former Soviet countries, namely Hungary, Estonia, Latvia, Lithuania, Slovakia, and Slovenia, for which data prior to the mid-90s is sparse. Additionally, we restrict the sample to observations prior to 2019 to not lose data on the United Kingdom, which discontinued emission reporting after leaving the European Union in 2020. For a direct comparison to our SDID estimations, we provide additional GSCM estimates based on the same restricted estimation sample in our online repository.

Estimation of health co-benefits. The estimation of health co-benefits was carried out with a two-step approach. First, we estimate a counterfactual pathway for regulated emissions in each country. More specifically, the equation explaining how the GSCM predicts counterfactual emissions changes is Equation [5] in the Report. Second, we leverage each country-specific counterfactual to compute an ATT for each country-regulated pair (denoted by τ_{it}). This approach allows us to capture the heterogeneity in abatement efforts (i) across different EU countries and (ii) over time. These individual ATTs are then averaged (cf., Equation [2] in the Report) to calculate average effects, which are presented in Figure 2 of the Report. Aggregate emission reductions are then computed by aggregating the absolute emissions reductions across periods and units, following the approach

by Bayer&Aklin (Ref. 9) (see also the beginning of 'results_table.r' in the online repository). These aggregate reductions are then multiplied with official EU-wide average cost estimates (see the following section) to yield the aggregate co-benefits. Next to the *Joint* estimate that captures effects of the EU ETS and concurrently tightened policies, in particular emission standards for LCPs, we also present a *Bounded* scenario in Figure 2 in the Brief Report, in which we subtract the estimated benefits from emission reductions of LCPs jointly regulated by the ETS and standards to bound the direct impact of the latter on aggregate benefits (see the SI section *Concurrent policies*).

Cost estimates for air pollution damages. The cost estimates provided by the German Environment Agency (EPA) (62, available here) used in our empirical analysis were derived from the EU NEEDS project. To the best of our knowledge, these provide the most comprehensive EU-wide cost estimates available in the time frame relevant for our historical analysis that are provided in official governmental guidance. Since the early 1990s, several large-scale European research initiatives have attempted to assess the external costs associated with energy production and other environmentally harmful activities (i.e., ExternE, CASES, MethodEx, and NEEDS), the results of which have been used in European cost-benefit analyses (63, 64). Drawing on previous efforts, the NEEDS project represents the latest research program conducted within this framework.

Specifically, NEEDS quantifies monetary damage costs associated with the emission of specific units of air pollutants through a methodology called the Impact Pathway Approach (IPA), which follows four steps. The first step involves identifying all pertinent emission sources, along with their associated technologies, within a geographical grid (50km x 50km), harnessing data from EMEP. The second step translates emissions into concentrations by modeling air pollutants, dispersion, and secondary pollution formation through chemical transformation mainly with the EMEP/MSC-West Eulerian model (65). The third step establishes the connection between pollution concentration and health impacts at the grid level, with the help of concentration-response functions (CRF) from the peer-reviewed literature (cf., Refs. 66, 67). Health impacts considered include mortality impacts as reflected by reduced life expectancy (expected years of life lost) and morbidity effects such as hospital admissions and medication use. Finally, the last step involves monetary valuation. The assessment of human health impacts has been conducted through a combination of a meta-analysis of existing values and own contingent valuation studies in 9 European countries (France, Spain, UK, Denmark, Germany, Switzerland, Czech Republic, Hungary, and Poland), drawing on the questionnaire from Ref. (68), that were later disseminated through a scientific publication (69).

The EU-wide cost estimates drawn from official guidance by the German EPA used in this Report here are adjusted for inflation using official data on the Harmonised Index on Consumer Prices (HICP), available from Eurostat. To determine the current price level, we average the 12 most recent HICP values (October 2022 to September 2023). In terms of damage sources, the cost estimates link the entirety of $PM_{2.5}$ damages to human health damages, which compares to a share of health-related damages of around 90% for SO_2 and 82% for NO_x (cf., NEEDS Research Stream 1b). The remaining shares of damages derive from non-human health impacts (biodiversity loss) as well as crop yield and material damages. Notably, the EU-wide average cost estimates from NEEDS are lower than the German-specific cost estimates provided by the German EPA, which is intuitive as German incomes are above average within Europe. Few other European countries provide official cost estimates, the UK being a prime exception. The UK Department for Environment Food and Rural Affairs provides damage cost guidance (available here), among others for SO_2, $PM_{2.5}$, and NO_x. Cost estimates differ somewhat from the ones provided by the German EPA and using the UK estimates instead in our *Main specification* would yield aggregate health co-benefits following the start of the EU ETS of around 447 billion Euros.

While using official cost estimates is a prudent choice, there are key caveats associated with these estimates, which reflect a broader limitation inherent in current government guidelines for the evaluation of air pollution damages in Europe. Relying on a single value to summarize population exposure, the CRF, and monetized health impacts (i.e., morbidity and mortality) assumes that the harm caused by a ton of SO_2, $PM_{2.5}$ or NO_x is the same irrespective of the location where it causes harm (after pollution dispersion and chemical transformation). This assumption similarly extends to studies employing CRFs from the literature to translate country-wide reductions in pollutant concentrations into quantifiable societal health benefits. While CRF-based approaches remain still common in the pollution-health literature (e.g., Ref. 70), it is essential to acknowledge that achieving a more precise estimation would ideally involve incorporating high-resolution spatially resolved damage estimates (71).

These caveats highlight the need to improve official governmental cost guidance on air pollution. There is important scope to advance our ballpark estimates in future studies that may (a) leverage granular stack-level data and directly model the atmospheric dispersion of pollutants across Europe as well as transboundary pollution (72) from beyond, (b) model population exposure using high-resolution data on population distributions, (c) explore heterogeneous, and potentially non-linear, dose-response relationships that can differ by pollutant and health outcomes as well as across locations and socio-economic characteristics, (d) examine how the monetary valuation of health benefits differs across locations and socio-economic characteristics, and (e) consider applying distributional weights for aggregating individual-level health benefits. The latter step would be consistent, for instance, with how the German EPA estimates the social cost of carbon (73) and is also recommended practice in the UK Treasury's Green Book (74). Arguably, these advances require a substantial interdisciplinary research effort, which is beyond the scope of this Brief Report. While we, here, focus on contributing a first ballpark estimate of the health co-benefits of treatment status since the start of the EU ETS—capturing the *joint* effect of the EU ETS and tightened emission standards and trying to *bound* the effects of emission standards—using aggregate emissions data and cost estimates, future work using dis-aggregated data promises to yield more refined and precise estimates of the co-benefits of the European Emission Trading System and other concurrent climate or pollution regulation policies.

References

1. EEA, Air pollutant emission inventory guidebook 2023: Technical guidance to prepare national emission inventories. *EEA Rep.* (2023).
2. R Calel, Adopt or innovate: Understanding technological responses to cap-and-trade. *Am. Econ. Journal: Econ. Policy* **12**, 170–201 (2020).
3. L Ntziachristos, Z Samaras, Passenger cars, light commercial trucks, heavy-duty vehicles including buses and motorcycles category. emep/eea emission inventory guidebook (2023).
4. Y Xu, Generalized synthetic control method: Causal inference with interactive fixed effects models. *Polit. Analysis* **25**, 57–76 (2017).
5. J Cui, C Wang, J Zhang, Y Zheng, The effectiveness of china's regional carbon market pilots in reducing firm emissions. *Proc. Natl. Acad. Sci.* **118**, e2109912118 (2021).
6. Y Hu, R Li, L Du, S Ren, J Chevallier, Could so2 and co2 emissions trading schemes achieve co-benefits of emissions reduction? *Energy Policy* **170**, 113252 (2022).
7. Y Zheng, R Tan, B Zhang, The joint impact of the carbon market on carbon emissions, energy mix, and copollutants. *Environ. Res. Lett.* **18**, 045007 (2023).
8. D Hernandez-Cortes, KC Meng, Do environmental markets cause environmental injustice? evidence from california's carbon market. *J. Public Econ.* **217**, 104786 (2023).
9. P Bayer, M Aklin, The european union emissions trading system reduced co2 emissions despite low prices. *Proc. Natl. Acad. Sci.* **117**, 8804–8812 (2020).
10. M Efthymiou, A Papatheodorou, Eu emissions trading scheme in aviation: Policy analysis and suggestions. *J. Clean. Prod.* **237**, 117734 (2019).
11. T Liu, L Ungar, K Kording, Quantifying causality in data science with quasi-experiments. *Nat. computational science* **1**, 24–32 (2021).
12. DB Rubin, Estimating causal effects of treatments in randomized and nonrandomized studies. *J. educational Psychol.* **66**, 688 (1974).
13. S Athey, GW Imbens, The state of applied econometrics: Causality and policy evaluation. *J. Econ. Perspectives* **31**, 3–32 (2017).
14. A Abadie, Using synthetic controls: Feasibility, data requirements, and methodological aspects. *J. Econ. Lit.* **59**, 391–425 (2021).
15. L Liu, Y Wang, Y Xu, A practical guide to counterfactual estimators for causal inference with time-series cross-sectional data. *Am. J. Polit. Sci.* (2022).
16. L Gobillon, T Magnac, Regional policy evaluation: Interactive fixed effects and synthetic controls. *Rev. Econ. Stat.* **98**, 535–551 (2016).
17. J Bai, Panel data models with interactive fixed effects. *Econometrica* **77**, 1229–1279 (2009).
18. Y Xu, L Liu, *gsynth: Generalized Synthetic Control Method*, (2022) R package version 1.2.1.
19. D Fullerton, E Muehlegger, Who bears the economic burdens of environmental regulations? *Rev. Environ. Econ. Policy* (2019).
20. J Sijm, K Neuhoff, Y Chen, Co2 cost pass-through and windfall profits in the power sector. *Clim. policy* **6**, 49–72 (2006).
21. N Fabra, M Reguant, Pass-through of emissions costs in electricity markets. *Am. Econ. Rev.* **104**, 2872–99 (2014).
22. B Hintermann, Pass-through of CO2 emission costs to hourly electricity prices in Germany. *J. Assoc. Environ. Resour. Econ.* **3**, 857–891 (2016).
23. M Grubb, P Drummond, Uk industrial electricty prices: competitiveness in a low carbon world. *Report* (2018).
24. AR Ferrara, L Giua, Indirect cost compensation under the eu ets: A firm-level analysis. *Energy Policy* **165**, 112989 (2022).
25. A Gerster, S Lamp, Energy tax exemptions and industrial production. (2023).
26. European Commission, Guidelines on certain state aid measures in the context of the greenhouse gas emission allowance trading scheme post-2012. *Off. J. Eur. Union* (2012).
27. P Basaglia, ET Isaksen, M Sato, Carbon pricing, compensation and competitiveness: lessons from uk manufacturing. *Grantham Res. Inst. on Clim. Chang. Environ. Work. Pap.* (2024).
28. J Colmer, R Martin, M Muûls, UJ Wagner, Does pricing carbon mitigate climate change? firm-level evidence from the european union emissions trading scheme. (2024).
29. M Fowlie, M Reguant, SP Ryan, Market-based emissions regulation and industry dynamics. *J. Polit. Econ.* **124**, 249–302 (2016).
30. KC Meng, Using a free permit rule to forecast the marginal abatement cost of proposed climate policy. *Am. Econ. Rev.* **107**, 748–84 (2017).
31. P Quirion, Historic versus output-based allocation of GHG tradable allowances: a comparison. *Clim. Policy* **9**, 575–592 (2009).
32. E Joltreau, K Sommerfeld, Why does emissions trading under the eu ets not affect firms' competitiveness? empirical findings from the literature. *Clim. policy* **19**, 453–471 (2019).
33. H Naegele, A Zaklan, Does the eu ets cause carbon leakage in european manufacturing? *J. Environ. Econ. Manag.* **93**, 125–147 (2019).
34. SF Verde, The impact of the eu emissions trading system on competitiveness and carbon leakage: the econometric evidence.

J. Econ. Surv. **34**, 320–343 (2020).
35. M Grubb, et al., Carbon leakage, consumption, and trade. *Annu. Rev. Environ. Resour.* **47**, 753–795 (2022).
36. A Dechezleprêtre, D Nachtigall, F Venmans, The joint impact of the european union emissions trading system on carbon emissions and economic performance. *J. Environ. Econ. Manag.* **118**, 102758 (2023).
37. A Dechezleprêtre, C Gennaioli, R Martin, M Muûls, T Stoerk, Searching for carbon leaks in multinational companies. *J. Environ. Econ. Manag.* **112**, 102601 (2022).
38. L Van Wortswinkel, T Dauwe, Emission scenarios for large combustion plants under the ied regime, (Eionet), Technical report (2018).
39. P Singhal, Are emission performance standards effective in pollution control? evidence from the eu's large combustion plant directive. (2021).
40. A Meyer, G Pac, Analyzing the characteristics of plants choosing to opt-out of the large combustion plant directive. *Util. Policy* **45**, 61–68 (2017).
41. M Leroutier, Carbon pricing and power sector decarbonization: Evidence from the uk. *J. Environ. Econ. Manag.* **111**, 102580 (2022).
42. European Commission, Report from the commission to the council and the european parliament on implementation of directive 2010/75/eu on industrial emissions, (European Commission, Brussels), Report COM(2021) 793 final (2021).
43. M Reynaert, Abatement strategies and the cost of environmental regulation: Emission standards on the european car market. *The Rev. Econ. Stud.* **88**, 454–488 (2021).
44. JJ Andersson, Carbon taxes and co2 emissions: Sweden as a case study. *Am. Econ. Journal: Econ. Policy* **11**, 1–30 (2019).
45. P Basaglia, SM Behr, MA Drupp, De-fueling externalities: Causal effects of fuel taxation and mediating mechanisms for delivering climate and health benefits. *CESifo Work. Pap.* (2023).
46. S Athey, M Bayati, N Doudchenko, G Imbens, K Khosravi, Matrix completion methods for causal panel data models. *J. Am. Stat. Assoc.* **116**, 1716–1730 (2021).
47. A Abadie, A Diamond, J Hainmueller, Comparative politics and the synthetic control method. *Am. J. Polit. Sci.* **59**, 495–510 (2015).
48. G Wood, TR Tyler, AV Papachristos, Procedural justice training reduces police use of force and complaints against officers. *Proc. Natl. Acad. Sci.* **117**, 9815–9821 (2020).
49. N Ratledge, G Cadamuro, B de la Cuesta, M Stigler, M Burke, Using machine learning to assess the livelihood impact of electricity access. *Nature* **611**, 491–495 (2022).
50. E Delarue, K Voorspools, W D'haeseleer, Fuel switching in the electricity sector under the eu ets: review and prospective. *J. Energy Eng.* **134**, 40–46 (2008).
51. R Calel, A Dechezleprêtre, Environmental policy and directed technological change: evidence from the european carbon market. *Rev. economics statistics* **98**, 173–191 (2016).
52. B Hintermann, S Peterson, W Rickels, Price and market behavior in phase ii of the eu ets: A review of the literature. *Rev. Environ. Econ. Policy* (2016).
53. IE Agency, The environmental case for natural gas (2017) Accessed on March 18, 2024.
54. U.S. Energy Information Administration, Natural gas and the environment (2022) Accessed on March 18, 2024.
55. M Crippa, et al., Ghg emissions of all world countries. *Publ. Off. Eur. Union: Luxemburg* (2023).
56. SK Grange, NJ Farren, AR Vaughan, J Davison, DC Carslaw, Post-dieselgate: evidence of nox emission reductions using on-road remote sensing. *Environ. science & technology letters* **7**, 382–387 (2020).
57. D Arkhangelsky, S Athey, DA Hirshberg, GW Imbens, S Wager, Synthetic difference-in-differences. *Am. Econ. Rev.* **111**, 4088–4118 (2021).
58. Y Gao, M Li, J Xue, Y Liu, Evaluation of effectiveness of china's carbon emissions trading scheme in carbon mitigation. *Energy Econ.* **90**, 104872 (2020).
59. K Tang, Y Zhou, X Liang, D Zhou, The effectiveness and heterogeneity of carbon emissions trading scheme in china. *Environ. Sci. Pollut. Res.* **28**, 17306–17318 (2021).
60. S Kranz, Synthetic difference-in-differences with time-varying covariates, Technical report (2022).
61. D Clarke, D Pailañir, S Athey, G Imbens, Synthetic difference in differences estimation (2023).
62. Umweltbundesamt, Best practice cost rates for air pollutants, traffic, power generation and heat production. appendix b of the "method convention 2.0 for the evaluation of environmental costs". (2012).
63. P Watkiss, S Pye, M Holland, Cafe cba: baseline analysis 2000 to 2020. *Rep. to Eur. Comm. DG Environ. Bruss.* (2005).
64. S De Bruyn, et al., Environmental prices handbook - eu 28 version, (CE Delft, The Netherlands), Ce delft report (2018).
65. D Simpson, et al., The emep msc-w chemical transport model–technical description. *Atmospheric Chem. Phys.* **12**, 7825–7865 (2012).
66. DE Abbey, et al., Long-term ambient concentrations of particulates and oxidants and development of chronic disease in a cohort of nonsmoking california residents. *Inhalation toxicology* **7**, 19–34 (1995).
67. CA Pope Iii, et al., Lung cancer, cardiopulmonary mortality, and long-term exposure to fine particulate air pollution. *Jama* **287**, 1132–1141 (2002).
68. A Krupnick, et al., Age, health and the willingness to pay for mortality risk reductions: a contingent valuation survey of ontario residents. *J. risk Uncertain.* **24**, 161–186 (2002).
69. B Desaigues, et al., Economic valuation of air pollution mortality: A 9-country contingent valuation survey of value of a

life year (voly). *Ecol. indicators* **11**, 902–910 (2011).

70. K Chen, M Wang, C Huang, PL Kinney, PT Anastas, Air pollution reduction and mortality benefit during the covid-19 outbreak in china. *The Lancet Planet. Heal.* **4**, e210–e212 (2020).
71. AL Goodkind, CW Tessum, JS Coggins, JD Hill, JD Marshall, Fine-scale damage estimates of particulate matter air pollution reveal opportunities for location-specific mitigation of emissions. *Proc. Natl. Acad. Sci.* **116**, 8775–8780 (2019).
72. G Shaddick, ML Thomas, P Mudu, G Ruggeri, S Gumy, Half the world's population are exposed to increasing air pollution. *NPJ Clim. Atmospheric Sci.* **3**, 23 (2020).
73. UBA, Methodological convention 3.0 for the assessment of environmental costs. *Umweltbundesamt* (2019).
74. HM Treasury, The green book: appraisal and evaluation in central government. (2023).
